# cGAS-STING pathway mediates activation of dendritic cell sensing of immunogenic tumors

**DOI:** 10.1007/s00018-024-05191-6

**Published:** 2024-03-21

**Authors:** Guohao Li, Xiangqian Zhao, Zuda Zheng, Hucheng Zhang, Yundi Wu, Yangkun Shen, Qi Chen

**Affiliations:** Fujian Key Laboratory of Innate Immune Biology, Biomedical Research Center of South China, College of Life Science, Fujian Normal University, Fuzhou, China

**Keywords:** cGAS-STING pathway, Type I interferon, DC, Tumor immune cycle

## Abstract

Type I interferons (IFN-I) play pivotal roles in tumor therapy for three decades, underscoring the critical importance of maintaining the integrity of the IFN-1 signaling pathway in radiotherapy, chemotherapy, targeted therapy, and immunotherapy. However, the specific mechanism by which IFN-I contributes to these therapies, particularly in terms of activating dendritic cells (DCs), remains unclear. Based on recent studies, aberrant DNA in the cytoplasm activates the cyclic GMP-AMP synthase (cGAS)- stimulator of interferon genes (STING) signaling pathway, which in turn produces IFN-I, which is essential for antiviral and anticancer immunity. Notably, STING can also enhance anticancer immunity by promoting autophagy, inflammation, and glycolysis in an IFN-I-independent manner. These research advancements contribute to our comprehension of the distinctions between IFN-I drugs and STING agonists in the context of oncology therapy and shed light on the challenges involved in developing STING agonist drugs. Thus, we aimed to summarize the novel mechanisms underlying cGAS-STING-IFN-I signal activation in DC-mediated antigen presentation and its role in the cancer immune cycle in this review.

## Introduction

Immune therapy harnesses the body's innate immune system to combat cancer, particularly through immune checkpoint inhibitors (ICIs) such as CTLA-4, PD-1/PD-L1, or LAG-3 [1, 2]. Immunotherapy prognosis is favorably connected with T-cell infiltration into the tumor, and the efficacy of ICIs depends on pre-existing anticancer T-cell responses [1, 3, 4]. To activate T-cell immune responses, dendritic cells (DCs) must first internalize and process tumor antigens. Subsequently, these captured antigens must be presented to T cells in the tumor-draining lymph nodes, contingent upon the major histocompatibility complexes (MHC-I and MHC-II). This initiation leads to the activation of effector T cells, which then recognize, bind to, and eliminate target cancer cells. This sequence of events is referred to as the tumor immune cycle [5]. The complete tumor immune cycle is crucial for initiating antitumor immunity, with the breakdown of tumor cells and antigen presentation marking the first steps in this cycle.

Tumor cells exhibit diverse forms of cell death, including apoptosis, necrosis, autophagy, and ferroptosis, elicited by physical, chemical, or biological interventions. In recent years, immunogenic cell death (ICD) has emerged as a novel mechanism in the tumor immune cycle. Subsequent to ICD, deceased tumor cells release damage-associated molecular pattern molecules (DAMPs) such as calreticulin (CRT), heat-shock proteins, high-mobility group box 1, and adenosine triphosphate. This process recruits and activates various immune cells, thereby augmenting the processing of tumor-associated antigens (TAAs) by DCs. In addition to the release of DAMPs, recent studies have revealed the pivotal role of type I interferon (IFN-I) release in promoting tumor ICD, with a deficiency in the IFN-I pathway impeding DC activation [6].

**Fig. 1:**
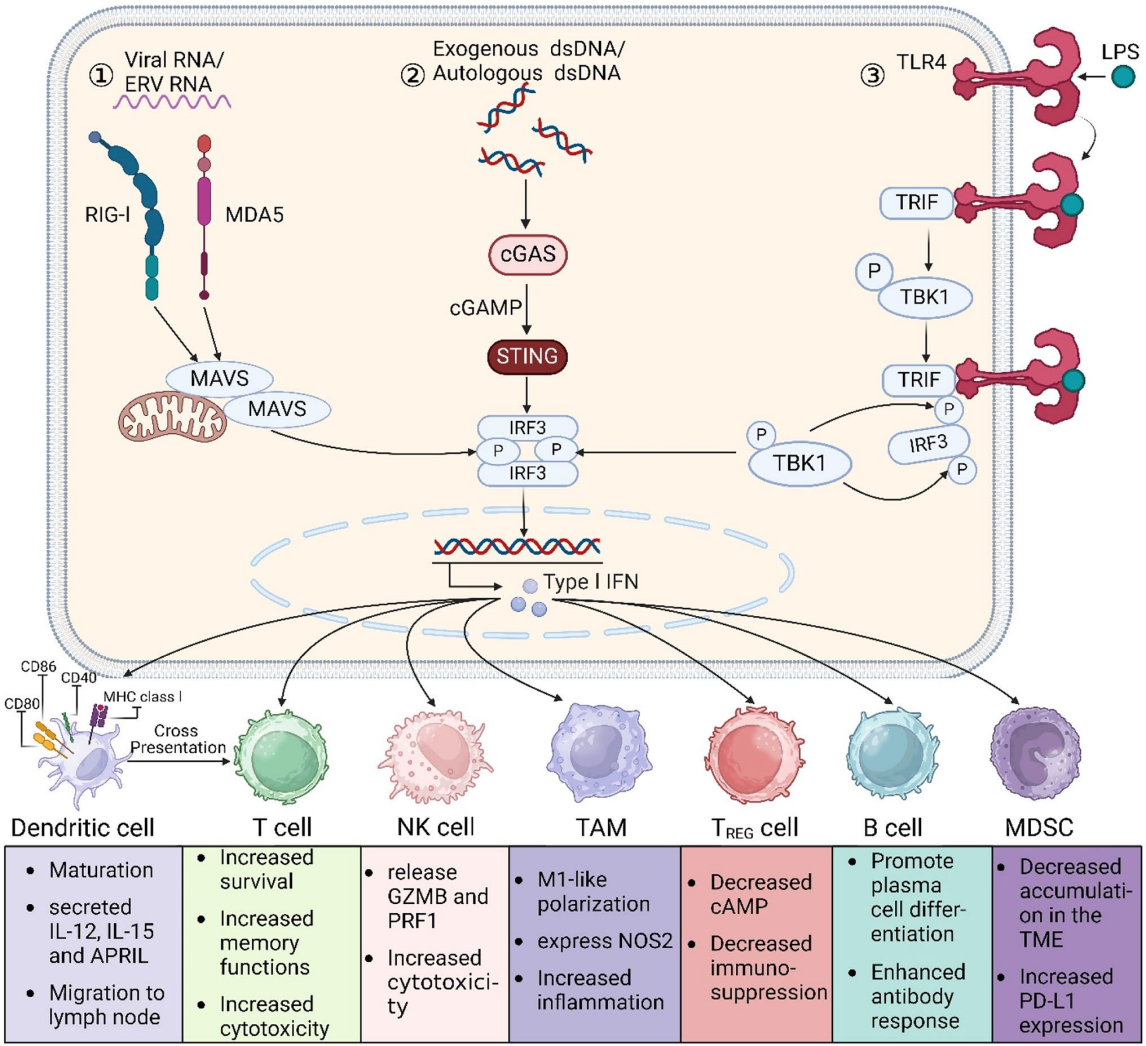
Immunomodulatory effects of IFN-I signaling**. ①**: RIG-I and MDA5, members of the PRR, are major cytoplasmic RNA sensors that, upon ligand binding, sequentially activate downstream axes to produce phosphorylated IRF3; **②**: STING is phosphorylated by TBK1 in response to DNA stimulation. DNA in the cytoplasm activates cGAS to produce the second messenger cGAMP, which then binds to STING and activates STING, and active STING then directly recruits and activates TBK1, resulting in phosphorylated IRF3; **③**: LPS stimulation activates TLR4, which in turn activates the adaptor protein MyD88 (not shown) and TRIF. TRIF activates TBK1, which in turn phosphorylates TRIF at consensus motifs. phosphorylated TRIF then recruits IRF3, which promotes phosphorylation of IRF3 by TBK1. Phosphorylated IRF3 dimerizes through the same positively charged surface. The IRF3 dimer then enters the nucleus and, together with NF-κB, initiates the production of type I interferons. After secretion, IFN-I interferons exert effects on various cells through different mechanisms. APRIL (official name TNFSF13), TNF superfamily member 13; *cAMP* cyclic AMP, *GZMB* granzyme B, *EMT* epithelial-to-mesenchymal transition, *MDSC* myeloid-derived suppressor cell, *NK* natural killer, *NOS2* nitric oxide synthase 2, *PRF1* perforin 1

IFN-I is a class of cytokines that can be triggered by receptors such as retinoic acid-inducible gene I (RIG-I), toll-like receptors (TLRs), and cGAS. They play a vital role in responding to bacteria, viruses, tumors, and cellular stress. Once activated, IFNs regulate the expression of numerous IFN-stimulated genes (ISGs) via the janus kinase-signal transducer and activator of transcription (JAK-STAT) pathway, thereby promoting antitumor immune responses. Numerous studies have shown that IFNs are a successful treatment for a variety of malignant cancers. Their primary effectiveness is in effectively stimulating DCs, which in turn promotes antigen cross-presentation and maturation. IFNs also exert an influence on other immune cells, including T cells, macrophages, NK cells, and regulatory T cells (Tregs), thereby regulating tumor immunity (Fig. 1). Clinical data indicate a positive correlation between elevated intratumor expression of IFNs or ISGs and improved prognosis [7]. As a result, using or activating IFNs has become a very promising approach in tumor immunotherapy.

The RIG-I/MDA5-MAVS and TLR-MyD88 pathways are activated by external viruses and bacteria, whereas the cGAS-STING pathway detects DNA from both external microorganisms and endogenous sources, such as nuclear and mitochondrial DNA [8]. When cGAS detects DNA, it generates cGAMP, which then activates STING and starts a cascade that results in the creation of IFN-I [9–11]. The cGAS-STING pathway enhances both innate and adaptive immunity by activating DCs, T cells, NK cells, and macrophages, with STING playing a crucial role as a transducer. Positive correlations between STING expression in tumors and immune cell infiltration have been observed in the cancer genome atlas program (TCGA) database [12]. STING can function in both IFN-dependent and -independent manners [13]. While research suggests that both cGAS-STING and IFN-I promote DC antigen cross-presentation, the specific mechanisms underlying DC activation remain unclear. Developing anticancer medications that target the cGAS-STING-IFN-I signaling axis requires an understanding of how cGAS-STING and IFN-I activate DCs.

In this review, we primarily address the methods by which the IFN-I-cGAS-STING signaling axis activates DCs in immunogenic tumors, especially considering the parallels and divergences between IFN and cGAS-STING activation processes. This evaluation is of paramount importance to gauge the profound impact of this pathway on innovative approaches to cancer treatment.

## DCs

**Fig. 2:**
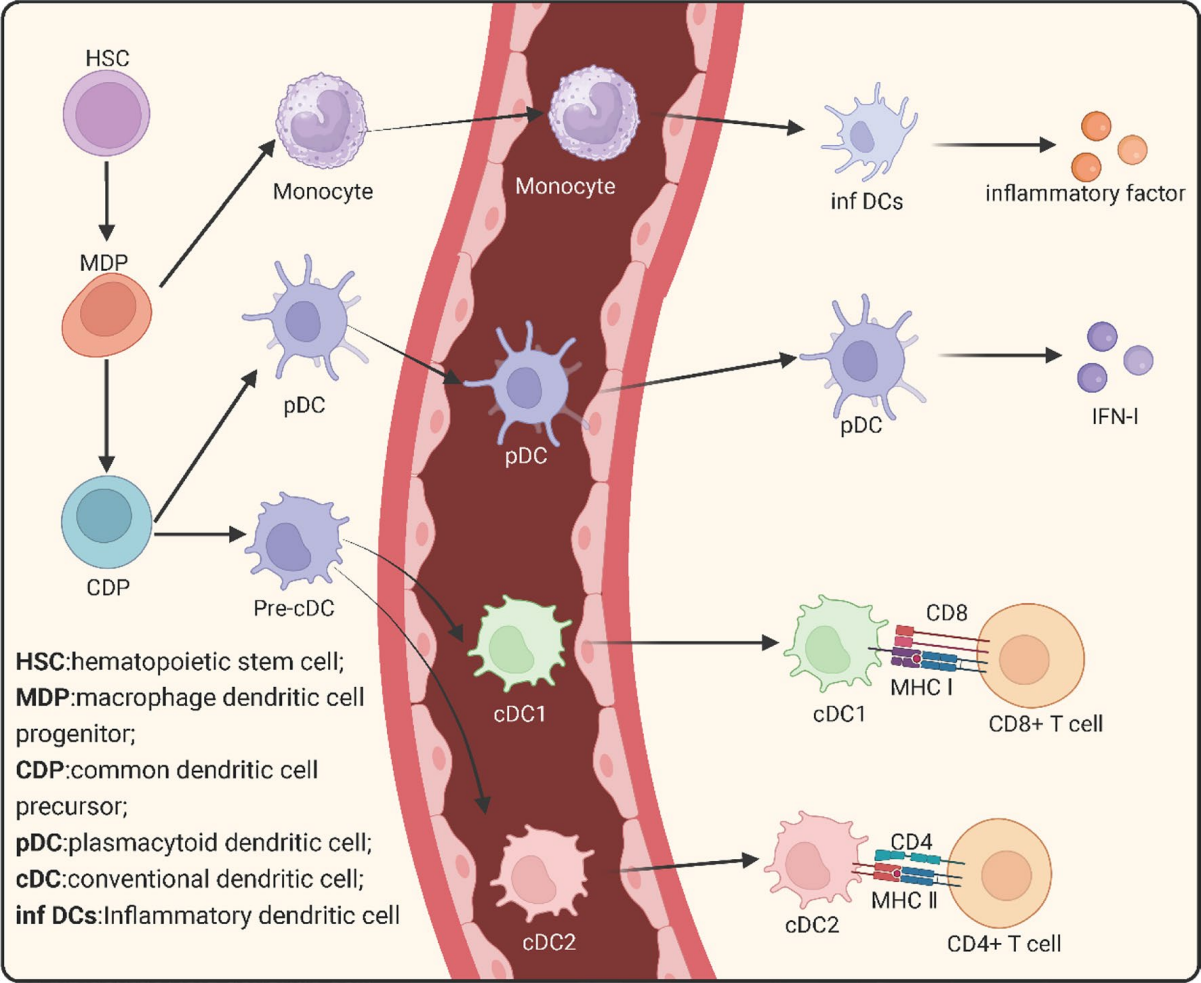
Classification and functional diagram of DC. In the bone marrow, hematopoietic stem cells (HSCs) give rise to macrophage-dendritic cell progenitors (MDPs). MDPs further differentiate into common dendritic cell progenitors (CDPs), which can to generate major dendritic cell (DC) subsets: plasmacytoid DCs (pDCs), conventional DC1 (cDC1), and conventional DC2 (cDC2). pDCs undergo terminal differentiation in the bone marrow, while pre-cDCs migrate through the bloodstream to lymphoid and non-lymphoid tissues, where they generate cDC1 and cDC2 subsets. On the other hand, MDPs differentiate into monocytes, and upon exposure to inflammatory cues, some monocytes differentiate into inflammatory dendritic cells under the influence of cytokines or chemokines

As a specialized class of antigen-presenting cells (APCs), DCs can be classified into three primary subtypes: conventional DCs (cDCs), plasmacytoid DCs (pDCs), and inflammatory DCs [14]. They play a pivotal role in engulfing tumor-specific antigens or tumor-associated antigens (TAAs) responsible for T cell-mediated tumor-specific cytotoxicity, thereby initiating the tumor immune cycle [15, 16]. Different DC subtypes possess unique mechanisms for antigen capturing and innate immune sensing, leading to variations in their abilities to acquire and process internalized antigens, produce cytokines, and activate T cells (Fig. 2) [15]. The composition of DCs within solid tumors varies depending on the tumor type, which in turn influences the antitumor T cell response [17]. Tumors with higher levels of DC infiltration often exhibit better responses to immunotherapies. Tumor-infiltrating DCs can exist in different functional states, which has significant implications for antitumor immune responses [18, 19]. In fact, numerous factors, including antigens, innate immune signals, adaptive immune cells, and environmental factors, can influence the state of DCs [20]. DCs recognize various pathogen-associated molecular patterns (PAMPs) and respond to the autocrine or paracrine release of cytokines independently of PAMPs for activation. Although interactions with CD4^+^ and CD8^+^ T cells can also induce DC activation [21–24], the main topics of this review are cDCs and pDCs, their functions in tumor immunity, and the ways in which they activate the innate immune system.

### The subsets of DCs

#### cDCs

The pre-cDCs, or precursors of cDCs, first enter the bloodstream from the bone marrow and then spread to different tissues. Within these tissues, pre-cDCs undergo differentiation into two distinct subtypes, namely type I (cDC1) and type II (cDC2). cDC1s are characterized by their exceptional antigen cross-presentation capabilities, effectively presenting extracellular antigens to cytotoxic CD8^+^ T cells via MHC-I. They play a major role in boosting anticancer immune responses and are excellent at fighting intracellular infections [25–28]. Although a few reports suggest that other cell types, such as macrophages, human pDCs, or mouse CD8α^+^CD11b^+^DCs, may also possess antigen cross-presentation capabilities [29, 30], the unique adaptation of this cross-presentation function primarily relies on the cDC1 subset, owing to its distinctive subcellular molecular mechanisms and vesicular transport [31]. On the other hand, cDC2s mainly use MHC-II to deliver antigens to distinct helper CD4^+^ T cell subsets, controlling how the body reacts to external infections.

While mouse and human DCs share high functional homology, they exhibit disparities in their molecular phenotypes [32]. Mouse cDC1s demonstrate elevated levels of CD11c, MHC-II, CD8α, and CD24 expression and typically express X-C motif chemokine receptor 1 (XCR1), CD141, C-type lectin receptor 9A (Clec9a), DEC-205, and CD103. Their development relies on a cascade of transcription factors, such as interferon regulatory factor 8 (IRF8), inhibitor of DNA binding 2 (Id2), and basic leucine zipper ATF-like transcription factor 3 (Batf3). In contrast, human cDCs development is governed by distinct transcription factors, including IRF8 and Batf3, in conjunction with the expression of CD141, Clec9a, and XCR1 [33–38]. Notably, human IRF8 can concurrently regulate the development of both cDC1s and cDC2s. Furthermore, both mouse and human cDC2s promote CD4^+^ T cell responses, albeit they possess relatively diminished capacities for antigen presentation to CD8^+^ T cells. A number of similar markers, such as CD11c, MHC-II, CD11b, and CD172a, are shared by cDC2s in both species.

#### pDCs

pDCs exhibit a distinctive secretory morphology reminiscent of plasma cells. Similar to cDCs, pDCs express the cytokine receptor Flt3 (CD135) and rely exclusively on its ligand, Flt3L, for their development. pDCs possess a unique capacity for robust IFN-I production, rendering them pivotal in antiviral and antitumor immunity. pDCs are predominantly located within lymphoid organs, where they circulate and typically constitute 0.1% to 0.5% of the total nucleated cells. Key markers expressed by human pDCs include CD303 (BDCA2) and CD123 (IL-3RA) [39–41].

#### Tumor-infiltrating DCs

Following tumorigenesis, DCs infiltrate into solid tumors, which are referred to as tumor-infiltrating DCs (TIDCs) within the TME. TIDCs encompass a diverse array of cell types, including cDCs and pDCs [42]. TIDCs are capable of presenting tumor antigens to T cells, thereby facilitating the tumor-immunity cycle. Reports indicate the clinical significance of TIDCs in various solid tumors, showing a strong correlation between TIDCs and clinical prognosis [43, 44]. However, TIDCs frequently show reduced antigen cross-presentation capacities [45]. A characteristic feature of immunologically dysfunctional TIDCs is an increased intracellular lipid content and augmented mitochondrial respiration. Within the TME, numerous immunosuppressive factors impede the maturation and antigen-presenting function of DCs function of DCs. For example, the expression of MHC class I and II molecules and their regulatory factors (such as CIITA) is reduced on TIDCs, along with downregulation of genes encoding ER-resident aminopeptidases (ERAP) and antigen processing-related transporters (TAP) [46]. The density and activation status of TIDCs can be determined by various molecules, with S-100/CD1a and CD83/DC-LAMP being the most widely used. Bell et al. confirmed that immature DCs expressing CD1a and langerin are distributed throughout tumors in breast cancer, while CD83^+^ and DC-LAMP^+^ (mature DCs) are localized in the peritumoral region [47]. Additionally, emerging studies have established lipid accumulation in dendritic cells (DCs) as a critical mechanism driving the dysfunction observed in TIDCs [48, 49]. The studies mentioned above indicate that to facilitate the completion of the tumor-immunity cycle, it is imperative not only to harness cytokines to attract an increased infiltration of DCs into the neoplastic tissue but also to promote antigen presentation and maturation of DCs effectively.

### Innate activation of DCs

The transmission of innate immunological signals is essential for triggering T and B cell responses. Central to this mechanism are DCs, whose activation by innate signals can induce their differentiation into immunogenic APCs and facilitate the transmission of information to lymphocytes. In this context, our main goals are to clarify the processes by which DCs are activated by innate immunological signals and investigate the impact of these processes on the immune response.

TLRs are well-known pattern recognition receptors (PRR) that can effectively activate DCs. A wide variety of TLR ligands function as adjuvants to enhance antigen cross-presentation [50, 51]. The actions of TLRs on DCs result in increased expression of MHC-peptide complexes, co-stimulatory molecules, and immune-regulatory cytokines, thereby fostering the initiation and activation of T cells. Except for TLR3, all TLRs activate TGF-β-activated kinase 1 via the adapter molecule MyD88 on DCs, subsequently activating the MAPK and NF-κB signaling pathways. This activation leads to the production of tumor necrosis factor-alpha (TNF-α), IL-12, and IL-6. cDC1s typically express most TLRs, except TLR5 and TLR7, with particularly high expression of TLR3. Human cDC1s express TLRs and secrete proinflammatory cytokines, including IL-12p70 and IFN-α, in response to infection-induced Th1 responses. Comparable to other steady-state DC subsets, cDC2s express a variety of TLRs but show higher levels of NOD-like receptor thermal protein domain associated proteins (NLRPs) and other inflammation-related signaling molecules, suggesting their functional specificity in identifying distinct danger signals. They usually secrete elevated levels of inflammatory cytokines, such as IL-6 and IL-8, and typically possess a mature phenotype in the lung-draining lymph nodes. They are distributed in the B cell zones of lymph nodes and are generally associated with antigen presentation to CD4^+^ T cells. While TLR1, 2, 4, 6, 8, and 9 are expressed in all examined DC subsets, the expression of TLR3, 5, and 7 varies significantly among DC subpopulations [52]. pDCs express high levels of intracellular nucleic acid-sensing TLRs, such as TLR7 and TLR9, which respectively recognize single-stranded RNA and unmethylated CpG-containing DNA sequences. pDCs respond to these nucleic acids by secreting a substantial amount of IFN-I and activating ISGs in other target cells [53]. Human pDCs do not express TLR4 and are unresponsive to lipopolysaccharide (LPS). On the other hand, while having low TLR4 expression, CD11c + DCs and monocyte-derived DCs (Mo-DCs) show considerable sensitivity to LPS activation [54, 55].

Despite TLR's potent ability to activate DCs, the specific activation mechanism remains unclear. Currently, it is believed that TLRs act on DCs by activating various members of the IRF family, thereby inducing IFN-I responses [56]. Notably, Hoshino et al. showed a significant decrease in the activation of mouse DCs when treated with TLR4 or TLR9 agonists in the absence of STAT-1 or IFNAR [57]. Another study revealed that IFN-I is a critical mediator induced by TLR7 agonist for cross-presentation [58]. Additionally, DCs from IFN-αβR^−/−^ mice exhibit a significant deficiency in stimulating T cells compared to DCs from wild-type (WT) mice. Therefore, the complete activation of DCs in response to TLR signals largely depends on the production of IFN-I.

Bacteria and viruses that invade the body can activate the RIG-I/MDA5-MAVS, cGAS-STING, and TLR-MyD88 pathways, which are the canonical pathways of IFN-I activation [59]. Seng-Ryong Woo et al. demonstrated that host cell STING and IRF3 play essential roles in initiating spontaneous CD8^+^ T cell responses against immunogenic tumors. This was observed in mice lacking MyD88, TLR4, TLR9, TRIF, P2X7R, MAVS, STING, and IRF3 [60]. Furthermore, the group illustrated that DNA derived from tumor cells activates IFN-β production and DCs via the cGAS, STING, and IRF3 pathways. Additionally, other studies have shown that the adaptor protein STING, rather than MyD88, is essential for the IFN-I-dependent antitumor effects mediated by radiation [61]. Based on these data, it appears that the DCs' cGAS-STING pathway is essential for the innate immune system to recognize DNA originating from tumors.

### DCs in cancer

Function-wise, cDC1s support CD8 + T cell responses and demonstrate strong antigen cross-presentation capacities. Additionally, cDC1s play a pivotal role in promoting tumor rejection responses. Studies have shown that mice lacking cDC1 expression in the tumor microenvironment (TME), either entirely or partially, exhibit a failure to generate antitumor responses [62]. Mice with a deficit in Batf3, for example, have impaired cDC1 formation and maturation, are unable to activate tumor-specific cytotoxic T cells, and are unable to generate effective antiviral responses [19, 63, 64]. In addition, recent findings underscore the importance of cDC1s in CD4^+^ T cell activation, with a few studies reporting that deficiency of cDC1s leads to a failure of CD4^+^ T cell activation [65, 66]. Although cDC1s are not very abundant in the TME, there is a positive association between their abundance and patient survival rates, which can be used as a measure of how well ICI therapy is working [18, 67].

cDC2s possess the capacity to elicit antitumor immunity [68]. They produce various cytokines, such as IL-10 and IL-23, and present TAAs to CD4^+^ T cells or transfer them to resident DCs within lymphoid tissues. cCD2s activate effector T cells, primarily Tregs, encompassing Th2 and Th17 cells responsible for immune tolerance. However, in both human and murine models, cDC2s are associated with a positive prognosis. For this reason, both cDC1s and cDC2s are beneficial for tumor immunity.

pDCs play an indispensable role in initiating antiviral immune responses. In mice, pDCs respond to CpG ODN and virus genomes with similar structures via TLR9 activation [69]. Given that pDCs are recognized as key players in the defense against viral infections [70], TLR9's primary physiological function likely involves the recognition of viral DNA. However, the role of pDCs in cancer is multifaceted. Conversely, the presence of pDCs within tumors has been linked to unfavorable outcomes in a number of malignancies, such as melanoma, ovarian cancer, breast cancer, and head and neck cancer [71–75]. Potential mechanisms may include pDCs inducing the co-stimulatory ligand inducible T cell co-stimulator, thereby promoting the expansion of Tregs [76]. Additionally, tumor-associated pDCs can activate Foxp3^+^ Tregs through indoleamine 2,3-dioxygenase (IDO) [77]. In patients with melanoma, pDCs express IDO, resulting in tryptophan depletion, impaired T cell function, and immune tolerance suggesting that pDCs can also elicit immunosuppressive responses. Furthermore, the infiltration of pDCs into tumors leads to reduced levels of IFN-α, sustaining the expansion of intratumoral Foxp3^+^ Tregs, thereby promoting immune tolerance and tumor progression.

Positively, pDCs have the ability tocan trigger immune-stimulatory reactions [78]. For example, a higher infiltration of pDCs has been significantly associated with non-progression and prolonged overall survival in patients with colon cancer. Functional deficits in pDCs have been observed across various cancer subtypes, characterized by attenuated responses to TLR7 or TLR9 activation and/or impaired IFN-I responses [74]. Furthermore, activation of pDCs has been demonstrated to induce antitumor immune responses, with several clinical trials employing pDCs demonstrating significant clinical benefits in human cancer treatment, underscoring the potential of pDCs in fostering antitumor immunity. Within the peritumoral region of primary melanoma, CD123^+^ pDCs closely interact with CD8^+^ T cells. Functional analyses have revealed that both human and murine pDCs can activate CD8^+^ T cells, leading to their differentiation into cytolytic and IFN-γ-producing effector T cells, thereby promoting tumor regression in vivo. In the TME, human CD2^high^ pDCs exhibit elevated levels of granzyme B, TRAIL, and lysozyme, which curtail tumor cell proliferation and mediate contact-dependent killing of tumor cells. Additionally, CD2^high^ pDCs efficiently secrete IL-12p70, stimulating naive T cells and resulting in T cell expansion and immune responses. Therefore, pDCs serve a dual role in tumor development, both promoting and inhibiting tumor growth. However, further investigation is required to elucidate the antigen presentation capabilities of pDCs.

In summary, DCs play a crucial role in maintaining immune homeostasis. However, the precise functions and significant contributions of individual DC subsets in maintaining immune equilibrium remain unclear. In addition to the aforementioned subtypes, DCs also exhibit other variations, including Langerhans cells (LCs) and Mo-DCs. Cross-presentation is a pivotal mechanism by which CD8^+^ T cells respond to exogenous antigens, especially those originating from deceased cells. Current research predominantly attributes the cross-presentation of tumor antigens to CD8α^+^ DCs. Nonetheless, intrinsically generated Mo-DCs can also efficiently participate in cross-presentation through a vesicular-dependent pathway [30]. Furthermore, unlike their murine counterparts, human pDCs have been reported to proficiently cross-present soluble cell-associated antigens [79]. Notably, a study showed that LCs adeptly cross-present antigens and promote the expansion of CD8^+^ T cells both in vitro and in vivo [58]. While most DC subtypes can engage in cross-presentation under specific experimental conditions, the optimal delivery of tumor cell-related antigens seems to be primarily limited to a subset of cDC1s [29]. Thus, further research is warranted to elucidate the specific roles of distinct DC subtypes in the TME during various stages of tumor development, which should establish a foundation for the advancement of cancer therapeutics.

## cGAS-STING-IFN-I axis mediates anti-tumor immune response

Numerous studies conducted since the discovery of cGAS have revealed a tight relationship between cancer immunotherapy and the cGAS-STING pathway [80–83]. Classical theories postulate that the cGAS-STING pathway triggers the IRF3-mediated IFN-I pathway and the NF-κB-mediated inflammatory pathway [84]. Recent studies have uncovered additional roles of the cGAS-STING pathway, including its involvement in autophagy, endoplasmic reticulum stress, and the regulation of cellular metabolism [85–88]. In this section, we provide an overview of both IFN-dependent and IFN-independent STING-mediated antitumor responses. We also investigate the known and possible mechanisms of DCs' response to STING.

### IFN-I-dependent cGAS-STING signaling-mediated tumor immune response

In recent years, mounting evidence suggests that, the integrity of the IFN-I signaling pathway is essential for radiotherapy, chemotherapy, targeted therapy, and immunotherapy [61, 89–91]. One significant reason for this is that normal intracellular expression of IFN-I serves as a form of immune surveillance, helping to prevent the development of tumors [92]. For instance, human breast cancer bone metastasis often occurs in conjunction with the presence of IFN-I-deficient cancer cells, attributed to reduced levels of IRF7 expression [93]. Reintroduction of IRF7 to IRF7-deficient tumor cells or supplementation with IFN-α effectively inhibits bone metastasis in a mouse model of breast cancer [93]. Furthermore, deficiency of either IFNAR1 or IFNAR2 increases the incidence of methylcholanthrene-induced fibrosarcoma in mice [94]. Ifnar1^−/−^ mice exhibit more severe chemically induced skin papilloma compared to their WT counterparts [95]. Therefore, the body's normal IFN-I signaling is frequently necessary for cancer immune surveillance.

As a critical component of intracellular DNA recognition, the cGAS-STING pathway primarily modulates tumor immune functions by eliciting the expression of IFN-I. For instance, in several mouse tumor models, tumors exhibit accelerated growth in mice deficient in cGAS or STING, concomitant with a diminished functionality of DCs [60, 61, 96]. Notably, the impairment in DC function was ameliorated by exogenous IFN-β treatment, thereby rescuing the cross-priming capability of cGAS or STING-deficient DCs [60].

Cellular DNA normally resides in the cell nucleus or mitochondria in a homeostatic state to avert spontaneous inflammatory reactions [97]. However, due to uncontrolled cell division and defective DNA repair, tumor cells often exhibit extensive DNA mutations and genomic instability. Damaged DNA or chromatin fragments are typically located within micronuclei (MN), and the membranes of these MN are highly prone to breakdown [98], increasing the likelihood of DNA leakage into the cytoplasm [99]. Studies have demonstrated that chemotherapy drugs can induce the production of immunogenic MN to initiate antitumor immune responses [100]. A significant portion of these MN co-localizes with cGAS, leading to the activation of the IFN-I response in these cells [101]. Therefore, the generation of mitotically dependent immunogenic MN can effectively activate the cGAS-STING pathway, thereby eliciting a systemic antitumor immune response.

However, many tumor cells employ immune evasion mechanisms by inhibiting this pathway. Therefore, effectively activating the cGAS-STING pathway within tumor cells emerges as a critical strategy to enhance the effectiveness of cancer treatment. Our prior research showed that the utilization of the demethylating drug zebularine induces the accumulation of DNA in the cytoplasm of tumor cells, enhances the expression of the STING gene, triggers the generation of IFN-I and the expression of ISGs, thereby increasing the sensitivity of tumor cells to the cGAS-STING pathway [102]. Subsequently, another study revealed that the reversal of STING methylation in mouse melanoma cell lines using a clinically available DNA methylation inhibitor, decitabine, ameliorates STING agonist-induced activation and IFN-I induction, thereby enhancing the CD8^+^ T-cell-dependent immune response that promotes tumor regression [103]. Several studies have reported that demethylating drugs effectively stimulate the activation of endogenous retrovirus (ERV) within cells, subsequently activating the cGAS-STING and RIG-I-MAVS pathways and enhancing the expression of IFN-I to bolster antitumor immune activity [104, 105].

Furthermore, the cGAS-STING pathway is widely expressed in immune cells such as DCs, macrophages, and T cells. The activation of this pathway effectively promotes the maturation of these immune cells. Notably, recent studies have revealed that a substantial number of tumor cells undergo cell death as a result of chemotherapy or radiotherapy, leading to the release of double-stranded DNA. DCs engulf this released DNA, which subsequently accumulates in their cytoplasm, thereby triggering the activation of the cGAS-STING signaling pathway. This activation leads to the robust production of IFN-I and inflammatory cytokines, such as TNF-α and IL-6. Thus, the intact cGAS-STING signaling pathway serves as a pivotal determinant not only for tumor cells but also for regulating the antitumor immune response in immune cells.

### IFN-I-independent STING-mediated tumor therapy

**Fig. 3:**
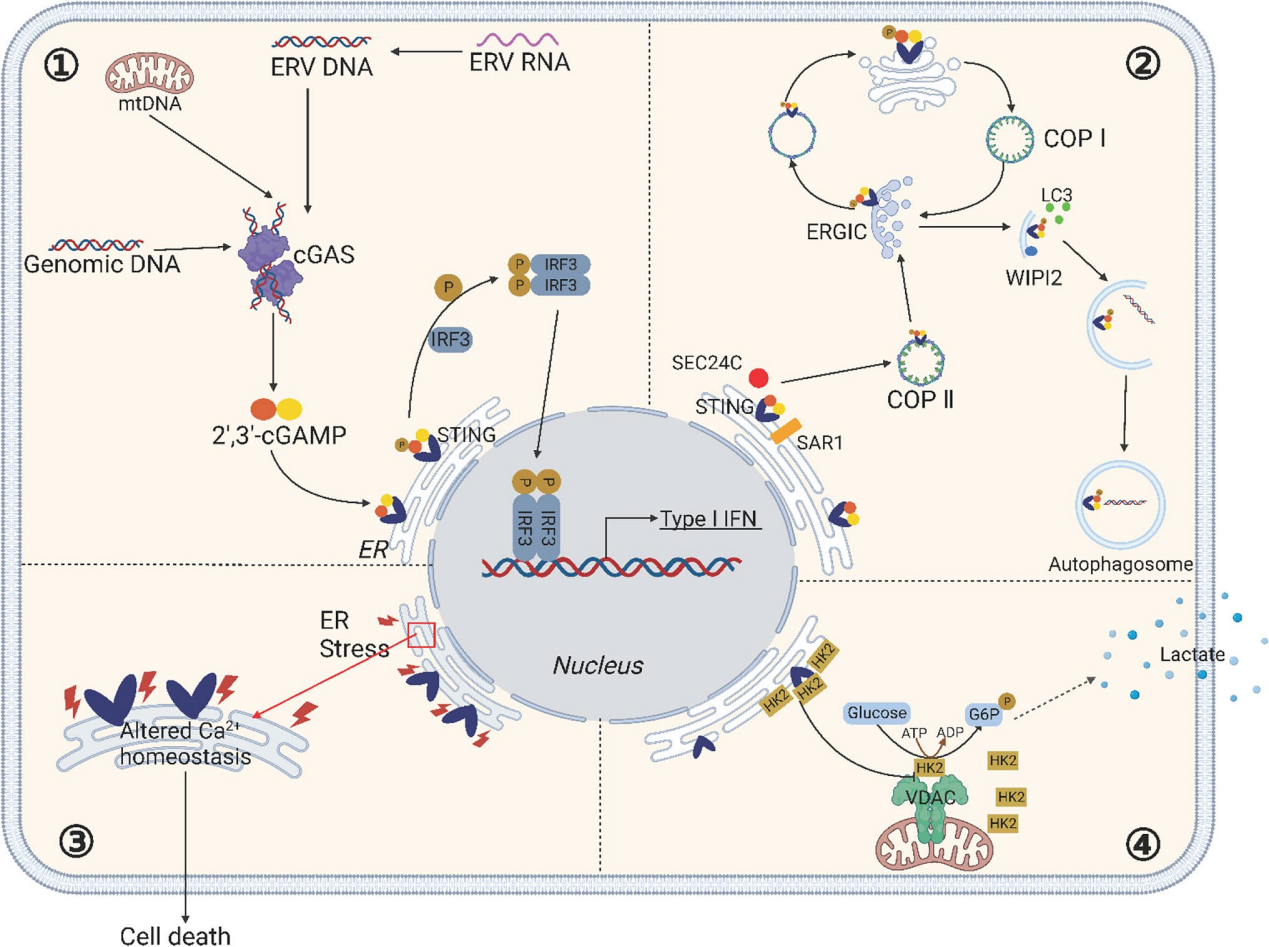
The Versatile Mechanisms of STING. ①cGAS-STING signaling pathway. The cGAS recognizes endogenous viral reverse-transcribed DNA or its DNA (from mitochondria or tumor cells). cGAS recognizes the DNA and then synthesizes the second messenger, 2′3'-cGAMP, which activates STING. The translocation of STING from the endoplasmic reticulum to the Golgi apparatus is a prerequisite for its involvement in downstream signaling and the regulation of IFN-I transcription. Activated STING recruits and activates TBK1, which phosphorylates IRF3. Subsequently, phosphorylated IRF3 forms a dimer and translocate to the nucleus, where it initiates the expression of immunomodulatory factors such as IFN-I. **②** STING and cellular autophagy. DNA from pathogens or damaged cells activates cGAS to synthesize cGAMP. cGAMP binds to STING and triggers the translocation of STING from the ER to the ERGIC and the Golgi apparatus, a process that is dependent on SAR1, SEC24C. The ERGIC, which contains STING, serves as a membrane source for LC3 recruit and lipolysis through a WIPI2-dependent mechanism. The LC3-positive membranes target DNA and pathogens to autophagosomes, which subsequently fuse with lysosomes. Accumulation of STING in lysosomes leads to lysosomal membrane permeabilization, which in turn leads to lysosome-dependent cell death. **③:** STING and endoplasmic reticulum stress Certain mutations in STING disrupt calcium homeostasis in T cells, which induces ER stress, leading to cell death. **④:** STING and cellular metabolism. STING binds to hexokinase 2 (HK2), the rate-limiting enzyme of glycolysis, and restricts the mitochondrial localization of HK2, thereby inhibiting its activity and, consequently, the process of glycolysis. ERV, Endogenous Retrovirus; ER, Endoplasmic reticulum; ERGIC, The ER-Golgi intermediate compart; COP I, coat protein I; COP II, coat protein II; VDAC, voltage-dependent anion channel

The antitumor response facilitated by STING extends beyond its conventional reliance on the cGAS-STING pathway and IFN-I. Recent studies have revealed that STING can influence tumor dynamics through various mechanisms such as IFN-independent mechanisms (Fig. 3) [80].

In 2009, Saitoh et al. reported the colocalization of STING and LC3 [106], implying an association between STING activation and cellular autophagy. In 2019, Xiang et al. systematically elucidated STING's role in autophagy [85]. They demonstrated that STING can activate autophagy through mechanisms independent of TBK1 activation and IFN-I induction, elucidating an ancient and highly conserved function of the cGAS-STING pathway in autophagy induction that predates the emergence of the IFN-I pathway in vertebrates. Subsequent studies revealed that STING activation not only induces classical autophagy but also triggers non-canonical autophagy pathways [107]. Additionally, STING can act as a proton channel mediating Golgi proton efflux, resulting in pH elevation and facilitating non-canonical autophagy and NLRP3 inflammasome activation [108]. Recent investigations have revealed that activated STING not only facilitates direct tumor cell death through the induction of autophagy but also mediates intercellular transfer to bolster anti-tumor immunity [109]. Mechanistically, this research has identified that RAB22A mediates STING-induced non-canonical autophagy. RAB22A-mediated atypical autophagosomes encapsulate activated STING, merging with RAB22A-positive early endosomes to form a novel structure, termed the Rafesome [109]. The Rafesome can then be secreted by the cell to extracellular space, forming RAB22A-induced extracellular vesicles (R-EVs). These STING-bearing R-EVs are competent to transfer between tumor cells, eliciting the secretion of IFNβ and thereby propelling anti-tumor immunity [109]. These results provide opportunities to investigate the possible involvement of STING in different downstream pathways related to different physiological processes. Such studies may significantly impact the development of STING-based treatment approaches.

The NF-κB-mediated inflammatory response constitutes a significant downstream consequence of STING activation. A recent investigation highlighted the pivotal role of the NF-κB-mediated inflammatory pathway in the antitumor effects of STING agonists [110]. In mice harboring the STING-∆CTT mutant, characterized by a C-terminal deletion of STING that interferes with its interaction with the downstream TBK1 protein, the activation of both IRF3 and NF-κB is compromised, while STING-mediated autophagy remains unaffected. Conversely, in STING-S365A mutant mice, a mutation that disrupts STING's ability to bind with IRF3, resulting in decreased IFN-I production, the activation of NF-κB and autophagic responses still occur. Notably, both WT and STING-S365A mutant mice exhibit robust antitumor immune responses when exposed to cGAMP in the LL2 lung cancer model, whereas such responses are conspicuously absent in ∆CTT mutant mice. These findings suggest that the antitumor responses induced by STING activation are contingent upon NF-κB-induced inflammation rather than autophagy.

STING also functions as an intracellular metabolic checkpoint, limiting aerobic glycolysis to promote antitumor immune responses [111]. Recently, Zhang et al. elucidated STING's role in curbing aerobic glycolysis, demonstrating that this function operates independently of its innate immune functions [112]. In this mechanical process, STING binds to the rate-limiting enzyme of glycolysis, hexokinase II (HK2), restraining HK2's mitochondrial localization, thereby inhibiting its activity and consequently suppressing glycolysis [112]. Thus, STING exerts inhibitory control over HK2 to curtail tumor aerobic glycolysis, ultimately fostering in vivo antitumor immunity. This discovery provides novel insights into the development of STING-based anticancer drugs and sheds light on why STING remains quiescent or is expressed at subdued levels in various cancer cells.

### cGAS-STING-IFN-I axis and DC activation

IFN-I exerts diverse effects on the activation and function of DCs. IFN-I can facilitate DC maturation by upregulating the expression of surface molecules such as CD80, CD83, CD86, and MHC class molecules, thereby enhancing their antigen presentation capacity. Additionally, IFN-I can induce DCs to express a spectrum of ISGs, further promoting the activation and proliferation of T cells. It is noteworthy that distinct DC subtypes may exhibit varying sensitivity to IFN-I, potentially resulting in heterogeneous responses.

IFN-I has been shown to facilitate cross-priming of CD8^+^ T cells to soluble antigens and plays a crucial role in activating DCs [113]. IFN-I treatment enhances antigen retention within DCs and decreases the acidification rate of intracellular endo-lysosomes [114]. A study validated that IFN-I promotes sustained antigen presence in CD8α^+^ DCs by regulating intracellular pH, consequently bolstering antigen cross-presentation by CD8α^+^ DCs [114]. Similar investigations have also demonstrated that IFN-secreting DCs significantly prolong the intracellular presence of antigen and decelerate intracellular acidification following OVA antigen uptake, consequently increasing MHC-I cross-presentation of the antigen [115]. Moreover, IFN-I promotes the transcription and translation of immunoproteasome subunits β1i (LMP2), β2i (MECL-1), and β5i (LMP7), thus enhancing peptide binding and presentation on MHC-I [116, 117]. Additionally, IFN-α not only promotes the localization of MHC-I to antigen storage compartments within DCs but also elevates the levels of MHC-I and MHC-II on the cell membrane [115]. Furthermore, IFN-α enhances the activation of CD8^+^ T cells by upregulating the surface expression of the stimulatory molecules CD40, CD80, and CD86 on DCs, as well as intracellular signaling in T cells [118].

As is well known, IFN-I primarily exerts its effects through downstream ISGs. IFN-I regulates the expression of numerous ISGs by activating the JAK-STAT pathway, thereby modulating the immune response [119]. To investigate the impact of ISGs on DC activity, a specific subset of DCs, characterized by a high expression of ISGs, has been identified as ISG^+^ DCs [113]. These ISG^+^ DCs lose their ability to induce effective antitumor T cell responses in *Ifnar1*^*−/−*^ mice. AXL, an IFN-induced engulfment receptor, has also been validated as a phenotypic marker for ISG^+^ DCs [120]. In colorectal cancer and melanoma, a small population of unactivated ISG^+^ DCs has been observed, which promote CD8^+^ T cells to attack tumor cells upon IFN-I treatment. While the specific ISGs responsible for mediating DC activation still need further identification, the expression analysis of various ISGs, including STAT1, MX dynamin-like GTPase 1 (MX1), C-X-C motif chemokine ligand (CXCL) 10, CXCL9, C–C motif chemokine ligand (CCL) 4, CCL5, HLA class I genes, absent in melanoma 2 (AIM2), and guanylate binding protein 1 (GBP1), in breast carcinoma has demonstrated predictive value for a favorable long-term outcome [121].

As previously mentioned, IFN-I can induce the maturation of DCs and supply tumor-specific antigens to CD4^+^ and protective CD8^+^ T cells [115]. Burnette et al. conducted experiments employing MyD88-deficient mice, TRIF-deficient mice, cGAS-deficient mice, and STING-deficient mice, and validated that IFN-I induced by radiotherapy can be attributed to the cGAS-STING pathway [61]. However, the robust antitumor effects observed in STING-induced IFN-I-independent functions suggest that other mechanisms, such as metabolic regulation and NF-κB-induced inflammatory functions, may also play significant roles in DC activation. Further research is required to fully elucidate the comprehensive regulation of STING-mediated antitumor effects.

## cGAS-STING-IFN-I signaling axis as a master regulator in the cancer immunity cycle

**Fig. 4:**
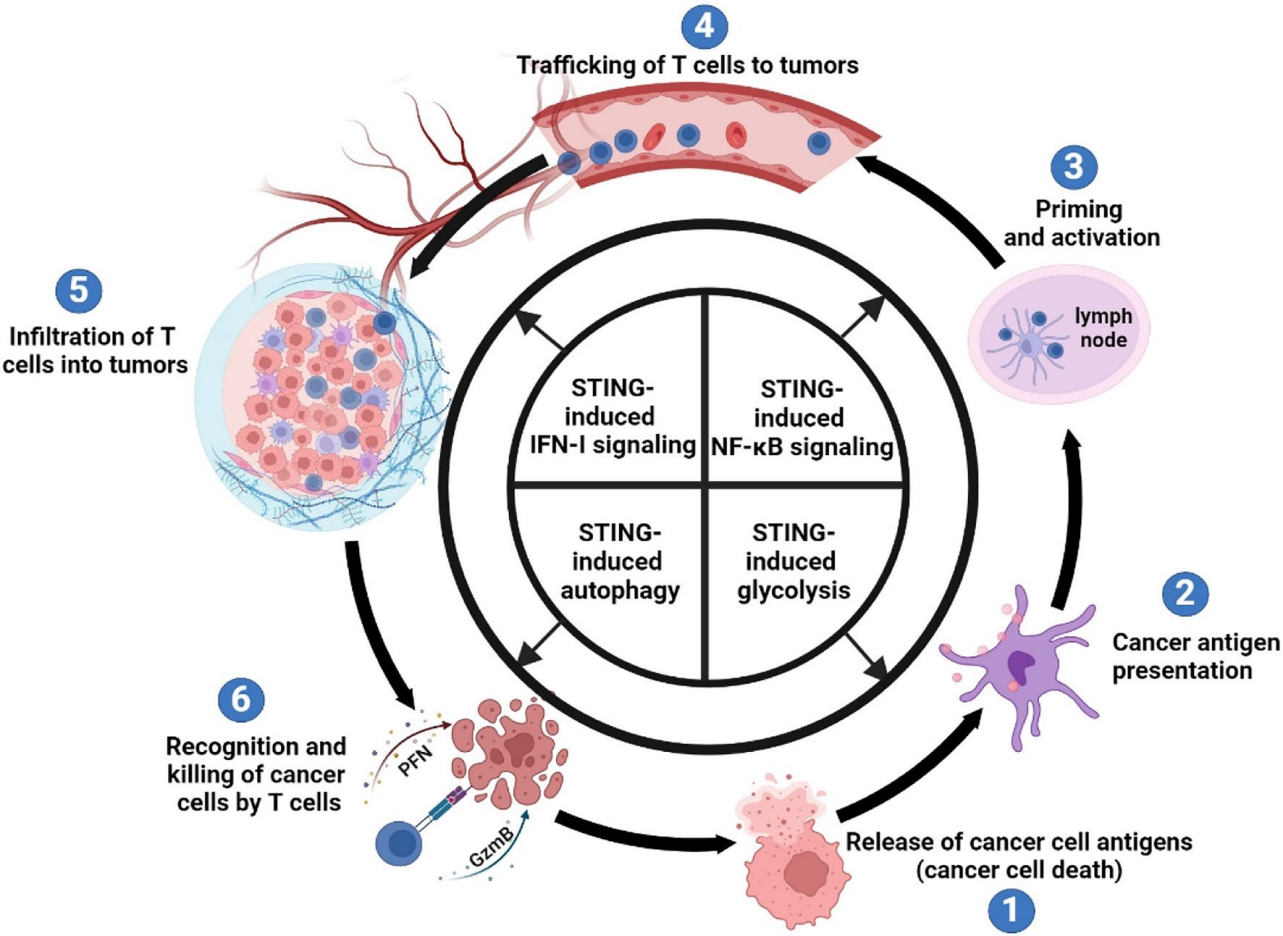
Activation of the cGAS-STING-IFN-I signaling axis positively regulates each step of the cancer-immunity cycle. The activation of the cGAS-STING pathway influences cancer immune responses through both IFN-I-dependent and IFN-I-independent mechanisms, involving autophagy, inflammation, and glycolysis

As a multifunctional signaling axis, cGAS-STING-IFN-I participates in nearly all stages of the tumor immune cycle, playing a pivotal role in the tumorigenesis process. Here, we will elucidate the roles and distinctions of IFN-I treatment and cGAS-STING pathway activation in the tumor immune cycle (Fig. 4).

One primary source of tumor antigens is tumor cell death. IFN-I directly inhibits tumor growth by inducing cell apoptosis, impeding cell cycle progression, and regulating tumor cell proliferation and differentiation [122, 123]. While the capacity of IFN-I to induce cell death in malignant cells has been recognized for over three decades, the underlying mechanisms of IFN-I action appear to be rather complex [124]. Nevertheless, IFN-I signaling has frequently been associated with apoptosis, involving widespread caspase activation [125]. Furthermore, IFN-I can inhibit tumor growth by suppressing angiogenesis. The inhibition of IFN-I on angiogenesis is primarily attributed to the upregulation of interferon-stimulated genes (ISGs) that suppress angiogenesis. ISGs such as STAT1, PML, IRF7, GBP1, ISG20, miR-106, and miR-16 exert a direct anti-angiogenic effect [121]. IFN-I also plays a crucial role in DC activation and antigen presentation. Recent studies indicate that IFN-I promotes the terminal differentiation and maturation of DCs, leading to increased surface expression of MHC-I, thereby enhancing immune recognition capabilities [118, 126–128]. This cytokine is essential for activating tumor-specific T cells and facilitating tumor elimination [129]. In the TME, CXCL9, CCL5, and CXCL10 are associated with CD8^+^ T cell infiltration [130]. IFN-I can induce the expression of CXCL9 and CXCL10, thereby regulating the trafficking and infiltration of CD8^+^ T cells [131].

Indeed, activation of the cGAS-STING pathway not only induces IFN-I for participation in the cancer immune cycle but also exhibits unique characteristics. For instance, while IFN-I induces cell apoptosis through various pathways, the cGAS-STING pathway can trigger IFN-independent autophagy in tumors. Moreover, the activation of the NF-κB pathway and glycolysis by the cGAS-STING pathway effectively promotes DC activation and immune cell infiltration in the TME.

In addition, a recent study [86] revealed that chromosomal instability (CIN) in tumor cells leads to sustained activation of the STING pathway. Notably, despite the persistent activation of the cGAS-STING pathway within tumor cells, it not only fails to promote the immune system's ability to eliminate these cells but also facilitates tumor migration. Specifically, this chronic activation of STING does not trigger the IFN-I response associated with tumor immune clearance. Instead, it initiates endoplasmic reticulum stress, leading to a pro-migratory TME and ultimately facilitating tumor immune evasion. The utilization of STING inhibitors has been shown to reduce metastasis in melanoma, breast cancer, and colorectal cancer driven by CIN [132]. This discovery establishes a theoretical basis for therapeutic strategies targeting CIN in tumor cells. Additionally, it underscores the intricate relationship between the activation status of STING in tumor cells and cancer treatment, connecting its role in immune and inflammatory functions with autophagy, metabolism, and the endoplasmic reticulum stress response.

STING agonists not only stimulate the production of IFN-I and the expression of ISGs but also activate cellular stress and death pathways [133]. In fact, within T cells, the activation of STING promotes T cell apoptosis [134]. Research has revealed the elevated production of the Noxa protein (a protein associated with cell death) following STING activation, resulting in cell apoptosis. STING induces T cell apoptosis by modulating endoplasmic reticulum stress and the unfolded protein response, leading to hyperactivation and cell death [135]. Through experiments involving STING S365A mutant mice, Wu et al. demonstrated that STING agonists robustly induce apoptosis in CD8^+^ T cells. Importantly, they showed that this effect occurs independently of the STING-induced IFN signaling pathway [136]. These findings indicate that tumor cells can trigger STING-mediated T cell apoptosis, facilitating immune evasion. Consequently, optimizing the dosage of STING agonists is crucial in tumor immunotherapy, striking a delicate balance between maximizing therapeutic benefits and preventing T cell apoptosis to enhance cancer treatment outcomes.

## Advances in the development of clinical drugs targeting the cGAS-STING-IFN I signaling axis

IFN-I has served as a potent immunomodulatory agent in clinical cancer therapy for over 30 years. However, its utility has been hampered by a short half-life and the onset of severe flu-like symptoms associated with its systemic administration. Recent years have witnessed a resurgence in the clinical application of IFN-I, owing to innovations such as antibody-conjugated IFN-I and the emergence of gene therapy. Additionally, the discovery of the cGAS-STING pathway has revealed novel avenues in cancer treatment, with STING agonists displaying remarkable potentials. STING agonists act as upstream signaling molecules for IFN-I, orchestrating a range of downstream responses, including IFN-I-independent autophagy, inflammation, endoplasmic reticulum stress, and metabolic regulation. Therefore, the development of STING agonists presents a broader scope within the realm of cancer therapy compared to IFN-I. Currently, drugs targeting the cGAS-STING-IFN-I signaling axis encompass diverse formats, such as protein drugs, small-molecule drugs, and gene therapy drugs (Table 1). We summarize the difficulties related to the cGAS-STING-IFN-I signaling axis and give an overview of the clinical advances made with these medications in the section that follows.

**Table 1:** STING agonists in partial clinical trials

STING agonists	Drug name	Condition	Species	Method of administration	Study Phase
DMXAA	Vadimezan	Mouse lung cancer, mesothelioma, human lung cancer and prostate cancer	Human, mouse	Intraperitoneal Injections	III
CDN	ADU-S100 [148]	Metastatic solid tumor or lymphoma	Human, mouse	Intertumoral Injections	I
	IMSA101	Breast cancer, malignant solid tumors	Human, mouse	Intertumoral Injections	I / IIa
	MK1454 [171]	Advanced/Metastatic Solid Tumors, Lymphoma, metastatic or unresectable recurrent squamous cell carcinoma of the head and neck	Human, mouse	Intertumoral Injections	II
	MK2118	Advanced/metastatic solid tumor or lymphoma	Human, mouse	Intertumoral Administration	I
	BI-1387446 [172]	Advanced, unresectable and or metastatic solid tumors	Human, mouse	Intertumoral Administration	I
	TAK-676 [173]	Advanced non-small cell lung cancer, triple-negative breast cancer, or squamous cell carcinoma of the head and neck	Human, mouse	Intravenous injection	I
	E-7766 [174]	High-risk non-muscle invasive bladder cancer	Human, mouse	Intertumoral Administration	I
	BMS-986301	Squamous cell carcinoma of the head and neck	Human, mouse	Intramuscular, intravenous and intra-tumoral administration	I
	SB-11285 [175]	Squamous cell carcinoma of the head and neck	Human, mouse	Intravenous injection	I / II
	DN-015089	advanced solid tumor	Human, mouse	Subcutaneous or Intertumoral Injections	Ia / Ib
	VB-85247 [176]	Non-muscle invasive bladder cancer	Mouse	Intravesical drip	Preclinical studies
	ONM-501	Advanced malignant solid tumors, lymphomas	Human、mouse	Subcutaneous or Intertumoral Injections	I
STING agonists—ADC	JAB-BX400	solid tumor	Human	None	Preclinical studies
	JAB-X1800	solid tumor	Human	None	Preclinical studies
	XMT-2056	gastric and breast cancer	Human, mouse	Systemic administration	Preclinical studies
	IMSA201	solid tumor	Human, mouse	none	Preclinical studies
engineering bacteria	SYNB1891 [177]	Melanoma, advanced solid tumors or lymphomas	Human, mouse	Intertumoral Administration	I
Others	diABZI STING agonist [178]	colorectal cancer	Mouse	Intravenous injection	Preclinical studies
	MSA-2 [179]	colorectal cancer	Mouse	Oral	Preclinical studies
	SR-717 [180]	Melanoma, colorectal cancer	Mouse	Intraperitoneal Injections	Preclinical studies
	SNX-281	Solid tumors, lymphomas	Human, mouse	Intravenous injection	I
	XMT-2056	HER2-positive breast cancer	Huma	Intravenous injection	I

### Progress in the clinical development of IFN-I drugs

IFN-I was initially approved for the high-dose treatment of various hematologic malignancies, including chronic lymphocytic leukemia [137]. It was believed that IFN-α's anti-angiogenic properties play a pivotal role in its antitumor efficacy by influencing tumor vasculature [138]. Subsequently, IFN-α received approval for the treatment of follicular non-Hodgkin's lymphoma, melanoma, and AIDS-related Kaposi's sarcoma [139]. However, the administration of high-dose IFN-α via intravenous route leads to acute symptoms, including flu-like reactions, thrombocytopenia, leukopenia, neutropenia, and depression. Therefore, the development of novel IFN-I agents and localized treatment approaches has emerged as a primary focus in the field of IFN drug development.

Polyethylene glycol IFN (PEG-IFN), designed to enhance IFN stability and extend its half-life, is commonly employed in the treatment of specific tumor types. In the context of chronic myeloid leukemia, combining PEG-IFN with targeted therapeutic drugs, such as imatinib, has proven effective in improving treatment outcomes [140]. Furthermore, conjugating IFN-I with antibodies represents another effective strategy for improving the efficacy of IFN-I therapy. For example, heterodimers of anti-CD20 antibodies and IFN-α fusion proteins (IGN002), which incorporate rituximab's antigen-binding sequences and full-length human IgG1/κ constant regions, are in development for the treatment of B cell lymphoma [141]. Yang et al. demonstrated the efficacy of antibody-IFN-β fusion proteins against various solid tumors in mice, targeting the epidermal growth factor receptor (EGFR) and Her2/neu [142]. Given the robust immune-activating properties of PD-L1 antibodies and IFN-α, the outlook for PD-L1 antibody-IFN-α fusion proteins in cancer treatment appears promising.

An illustrative case involved the incorporation of IFN-β into the VSV virus for the treatment of liver cancer in mice, rats, and rhesus monkeys [143]. Furthermore, in a phase IB clinical trial utilizing an oncolytic virus, the G207 vector derived from HSV-1, featuring a γ34.5 deletion, demonstrated the ability to induce heightened inflammatory responses and increased ISG expression [144]. Therefore, enhancing the expression of IFN-β during oncolytic virus therapy may more effectively reinforce the antitumor immune processes mediated by the virus. Nevertheless, given that IFN-I can effectively activate antiviral immune responses, the integration of IFN-I into viral particles for gene therapy still presents daunting challenges.

### Progress in the clinical development of STING-agonist drugs

The flavone acetic acid derivative DMXAA, known for its vascular-disrupting capabilities on established tumor vasculature, induces tumor cell death without affecting normal tissues [145]. Subsequently, DMXAA was identified as an agonist for STING. In murine tumor experiments, DMXAA demonstrated promise, reducing mouse fibrosarcoma size and increasing specific T cell counts [146]. In a phase II clinical trial, a combination therapy of DMXAA with carboplatin (CBP) and paclitaxel exhibited superior efficacy in treating advanced non-small cell lung cancer compared to CBP and paclitaxel alone [147]. However, these favorable outcomes were not replicated in a phase III clinical trial, leading to its discontinuation. This discrepancy arises from the species-specific nature of DMXAA, as it is not sensitive to human STING activation. Therefore, the primary focus in developing STING agonists has shifted towards human STING activation.

ADU-S100/MIW815 is a synthetic cyclic dinucleotide that mimics the natural ligand required for the activation of human STING [148]. Preclinical studies have shown the induction of tumor-specific CD8^+^ T cells through intratumoral administration of ADU-S100 [148]. However, clinical research has indicated that ADU-S100 as a monotherapy exhibits limited efficacy. Nonetheless, when combined with PD-1 antibody drugs, it demonstrates significant antitumor effects [149]. In a phase Ib clinical trial (NCT03172936) assessing the effectiveness and safety of spartalizumab in combination with ADU-S100 for patients with advanced solid tumors or lymphomas, only five patients exhibited significant responses, comprising two complete responses and three partial responses, resulting in a 9.4% response rate. Additionally, only 6 out of 30 patients achieved stable disease (SD) in the monthly injection group [149]. Another STING agonist, MK-1454, did not lead to any remissions when used as monotherapy, as indicated by preliminary data presented at the 2018 ESMO conference (Abstract 5475). However, when used in combination with the PD-1 inhibitor pembrolizumab, a partial response rate of 24% was observed. Preclinical results for STING agonists, such as SB11285 and MSA-2, have demonstrated significant antitumor effects. However, more clinical studies are necessary.

These clinical findings suggest that the therapeutic efficacy of STING agonists as monotherapy in cancer treatment is limited for several reasons. Firstly, a complete tumor immune cycle necessitates the release of antigens, a process in which STING agonists alone exhibit restricted effectiveness. Secondly, the key step in activating antitumor immunity involves alleviating immune checkpoint inhibition on T cells. However, STING agonists not only fail to downregulate the expression of PD-1/PD-L1 but also significantly enhance the expression of multiple immune checkpoints [150]. Thirdly, in clinical trials, achieving optimal therapeutic outcomes with low doses of STING agonists poses challenges, while high doses may lead to severe adverse reactions. In addition to common side effects, such as injection site pain, fever, fatigue, and itching, high-dose STING agonists induce apoptosis in CD8^+^ T cells, compromising the immune response. These challenges complicate safety assessments during regulatory scrutiny. Lastly, prolonged exposure to high doses of STING agonists can induce tolerance in target cells, thereby promoting tumor formation. Therefore, it is imperative to investigate the optimal dosage and duration of STING agonist administration.

In addition, the issues of drug delivery and off-target effects present significant challenges in the development of small-molecule STING agonists. A recent study involved the genetic engineering of bacteria for the purpose of delivering STING agonists [151]. *Escherichia coli* Nissle 1917 carrying the diadenosine cyclase gene, when injected into melanoma mouse tumors, was observed within the tumor mass but not in the subcutaneous space surrounding the tumor. This localization contributed to a reduction in tumor size [151]. Additionally, in the context of the increasing utilization of ADC drugs, STING agonists are now being employed as conjugate drugs for ADC. The STING agonist ADC employs antibodies to facilitate the targeted delivery of small-molecule immunostimulants to the TME and their subsequent localized release. This approach addresses the challenge of achieving a narrow safety window for immunostimulant system delivery while simultaneously enhancing the effectiveness of antitumor therapy [152]. An ADC has been synthesized that combines a STING agonist with an antibody targeting the EGFR. This resulting ADC effectively activated immune cells in vitro and demonstrated potent therapeutic effects with minimal toxicity in a syngeneic mouse tumor model [152].

Furthermore, the application of STING agonists within cancer vaccines may represent a novel approach to circumvent the systemic administration of these agents. STINGVAX, a combination cancer vaccine targeting the STING pathway, combines cyclic dinucleotides (CDNs) with tumor antigens and GM-CSF [153]. Relevant experiments have shown that it can activate dendritic cells (DCs) in the draining lymph nodes (DLNs) in vivo, and its ability to activate DCs is stronger than lipopolysaccharide (LPS). Experimental results from mice with STING gene mutations and IFNαR − / − mice demonstrate that the anti-tumor effect of STINGVAX requires functional STING and type I interferon involvement [153]. Tumor-derived microvesicles (T-MPs) represent another type of tumor vaccine containing specific proteins and nucleic acids from parental cells [154]. Research has indicated that T-MPs influence the immunogenic phenotype of DCs and produce potent anti-tumor immunity in processes of prevention and therapy by mediating antigen transfer from macrophages to DCs and inducing type I interferon production via the cGAS/STING pathway. Additionally, evidence suggests that microvesicles from allogeneic tumor cell lines appear to harbor shared tumor antigens that can be cross-presented by host DCs, providing potential simplification for clinical application of T-MP-based vaccines [154]. DNA@CaCO_3_, a cGAS-STING agonist synthesized by simple biomineralization growth of dsDNA-calcium carbonate (CaCO_3_) microparticles, activate DCs through intracellular cGAS-STING pathways by promoting dsDNA escape from endosomes, thereby inducing their maturation and activation to initiate an immune response [155].

Another challenge pertains to the presence of single-nucleotide polymorphisms within the STING gene, i.e., there exist five primary STING variant types in the human population: R232 (57.9%), HAQ (20.4%), R232H (13.7%), AQ (5.2%), and R293Q (1.5%). Research indicates that these human STING variants exhibit varying responses to STING agonists. Furthermore, the prevalence of these variants displays significant disparities among distinct population groups. For instance, the HAQ variant is prevalent among East Asians but rare among Africans [156]. Therefore, it becomes imperative to account for the distribution of STING variants within the population when selecting patients for clinical trials.

In summary, these findings underscore the crucial role played by the STING-mediated IFN-I-dependent signaling pathway in antitumor immune responses. As our understanding of these strategies advances, they offer promising avenues for further research and application in the field of cancer immunotherapy.

## Discussion

In recent years, significant progress has been achieved in the field of cancer treatment, with a particular emphasis on T cells, including PD-1 antibodies, CAR-T, and TILs. The essential role played by T cells in recognizing and eradicating tumors is crucial. However, the activation of DCs, a critical step in T cell-mediated immunity, is frequently impeded by the highly immunosuppressive TME in most solid tumors [157, 158]. Tumor cells often disrupt the normal process of antigen presentation by inhibiting the maturation of DCs, resulting in the failure of T cell activation and, ultimately, the development of immune tolerance [159, 160]. Hence, the effective presentation of antigens through the activation of DCs is of paramount importance for the establishment of T cell-specific immunity.

Several in vivo studies have indicated that cDCs are indispensable in antigen cross-presentation, whereas pDCs and LCs exhibit lowered effectiveness compared to cDCs [29, 63]. When danger signals are detected, cDCs become activated by recognizing PAMPs or DAMPs [161]. Activated cDCs capture antigens and migrate to lymph nodes, where they present these antigens to both CD8^+^ and CD4^+^ T cells, initiating the immune response [37, 162]. Current research on DCs extends beyond cDCs alone, with animal experiments typically involving DCs derived from bone marrow. The activation of DCs often relies on innate immune signals, primarily mediated by cytokines. In preclinical experiments, cytokines such as IFN-α, GM-CSF, IL-2, IL-12, IL-15, and IL-21 have demonstrated effective treatment of mouse tumors. When investigating factors contributing to DC maturation, Thomas Luft et al. discovered that only IFN-α or IFN-β accelerated maturation, with most cells acquiring mature DC characteristics within three days [118]. However, the precise mechanism of IFN-I-mediated activation of DCs is not fully understood.

In recent years, research into the cGAS-STING signaling pathway has unveiled a close association between IFN-I and cGAS-STING activation in DC activation. While they share multiple functions, they exhibit distinct mechanisms. Both IFN-I and cGAS-STING agonists induce cellular apoptosis, but IFNs primarily affect tumor cells, whereas STING agonists impact various immune cell types, including T and B cells. Additionally, IFN-I activation in DCs leads to the expression of ISGs, which activate immune cells. In contrast, STING agonists not only induce IFN-I but also prompt DCs to release cytokines such as TNF-α and IL-6, thereby comprehensively activating the immune response. As an upstream signal for IFN-Is, STING agonists also mediate IFN-I-independent responses, including autophagy, inflammation, endoplasmic reticulum stress, and metabolic regulation. Consequently, the development of STING agonists holds broader potential in cancer therapy compared to IFN-I.

IFN-I encompasses a group of proteins, including IFN-α, IFN-β, and less explored variants such as IFN-ε, IFN-κ, and IFN-ω. For drug development targeting this pathway, IFN-α2 has been utilized for nearly 30 years, while IFN-β and other IFN-α types are still in the investigational phase. Studies have revealed that various IFN-α subtypes, particularly IFN-α14, exhibit diverse abilities to stimulate downstream pathways. IFN-α14 has demonstrated significant advantages over IFN-α2 in combating HBV infection, implying potential divergent antitumor effects among various IFN-α variants. While IFN-α and IFN-β share similar mechanisms in the treatment of tumors, IFN-β exhibits a higher affinity for IFNAR1/IFNAR2 receptors [163]. Consequently, compared to various IFN-α isoforms, IFN-β can modulate cellular functions at lower concentrations. The findings suggest that the development of targeted therapies for specific IFN-α subtypes or the formulation of enhanced IFN-beta preparations may represent novel strategies for leveraging Type I interferons in the treatment of cancer.

Relative to IFN-I, STING agonists possess the capacity to modulate an extensive array of downstream pathways. Presently, various STING agonists have demonstrated promising results in preclinical animal models, showing significant tumor burden reduction and immune activation. Moreover, as a potent activator of the cGAS-STING pathway, manganese ions can significantly enhance the host's anti-tumor immune function, effectively reducing the cost of tumor immune therapy [164]. This presents a novel avenue for drug development in STING agonists. Nonetheless, the advancement of STING agonists is met with considerable challenges, particularly concerning the potential damage to T cells, which is of paramount importance. This underscores the necessity for precision in the development of STING agonists, emphasizing their specificity to certain cell types.

Furthermore, recent studies have demonstrated that STING agonists may promote the development of tumors in some cancer types [165, 166]. On one hand, STING activation can promote chronic inflammation, thereby driving cutaneous carcinogenesis. Compared to wild-type mice, STING-deficient mice exhibit resistance to mutagen-induced skin tumorigenesis [165]. On the other hand, activation of STING supports tumor development by facilitating the infiltration of Tregs into the tumor microenvironment, upregulation of immune checkpoint molecules, and secretion of IL-10, which collectively serve to suppress T cell activity [166–168]. Overall, STING facilitates the growth of tumors by promoting chronic inflammation, which lays the groundwork for an immunosuppressive tumor microenvironment. It is noteworthy that chronic inflammation is a prolonged process in tumorigenesis, suggesting that sustained activation of STING at sites of inflammation can lead to tumor development. However, in the context of cancer therapy, the application of STING agonists constitutes a short-term, high-dose treatment strategy capable of effectively initiating the tumor-immunity cycle and promoting tumor regression highlighting the necessity for further comprehensive research and consideration in the development of small-molecule STING agonists. Fortunately, preclinical research combining ADC medications with gene-based strategies and STING agonists has produced encouraging outcomes, suggesting a new path for STING agonist development [152]. The development of cancer vaccines targeting the cGAS-STING-IFN I signaling pathway represents another direction in drug development. However, the high incidence of adverse events such as nausea, fatigue, and flu-like symptoms during DC vaccine use still requires further improvement [169, 170].

In summary, both IFN-I and STING agonists play essential roles in activating DCs and promoting immune responses. However, they exhibit distinct mechanisms and functions. A comprehensive understanding of these commonalities and disparities is vital for comprehending immune regulation and disease treatment mechanisms. As our comprehension advances, these insights offer potential for further research and applications in the field of cancer immunotherapy.

## Data Availability

This review article did not look at any new data. Only results published in previous studies and identified in the reference list below were used.
